# Effect of transplanted oligodendrocyte precursor cells derived from inflammatory and non-inflammatory microenvironment on remyelination in a chronic cuprizone model

**DOI:** 10.1371/journal.pone.0343039

**Published:** 2026-02-18

**Authors:** Hoda Akbari, Iraj Ragerdi-Kashani, Farzaneh Rezaei-Yazdi, Parichehr Pasbakhsh

**Affiliations:** Department of Anatomical Sciences, School of Medicine, Tehran University of Medical Sciences, Tehran, Iran; Suez Canal University Faculty of Medicine, EGYPT

## Abstract

**Introduction:**

Multiple sclerosis is a chronic demyelinating disease of the central nervous system. Transplantation of oligodendrocyte progenitor cells (OPCs) is a promising approach to enhance remyelination; however, the influence of the OPCs’ microenvironmental origin on their therapeutic efficacy remains unclear. This study compared the remyelinating capacity of OPCs isolated from inflammatory (lipopolysaccharide) and non-inflammatory (cuprizone) microenvironments after transplanting into the corpus callosum and examined their effects on extracellular matrix chondroitin sulfate proteoglycans (CSPGs).

**Methods:**

OPCs were isolated from two microenvironments and characterized by immunocytochemistry and RT-qPCR. After transplanting, OPC homing, remyelination, gene expression, and CSPG levels were evaluated using DiI labeling, LFB staining, RT-qPCR, and immunofluorescence, respectively.

**Results:**

Severe demyelination exhibited in the cuprizone group compared with healthy controls (p < 0.001) by Luxol fast blue staining. Myelin content significantly increased in both transplating OPCs groups (p < 0.001), with a higher impact observed in mice received OPCs isolated from cuprizone as compared with lipopolysaccharide (p < 0.001). Also, RT-qPCR analysis exhibited significantly reduced MBP expression in the cuprizone group, whereas was significantly increased after OPC transplantation, particularly in the cuprizone-derived OPC group (p < 0.001), whereas a lower increased with lipopolysaccharide-derived OPCs (p < 0.01). MOG expression exhibited a same pattern, with a significantly increase in the cuprizone-derived OPC group compared with both the cuprizone and lipopolysaccharide-derived OPC groups (p < 0.001). Additionally, Immunofluorescence analysis exhibited increasing CSPG4 levels in the cuprizone group, but significantly reduced after OPC transplantation (p < 0.001). Notably, in the cuprizone-derived OPC group higher reduction of CSPG4 levels observed compare with in the lipopolysaccharide-derived OPC group (p < 0.001).

**Conclusion:**

OPC transplantation improves remyelination and reduces the CSPG level, but the effectiveness is more related to the previous history of the OPC isolation microenvironment and the new donor.

## Introduction

Multiple sclerosis (MS) is the prevalent neurodegenerative disease distinguished by severe demyelination, oligodendrocyte (OL) death, and axon damage within the central nervous system (CNS), which results in neurological disabilities in youthful individuals [1,2]. Research has revealed novel cell-based therapy strategies to avoid irreversible disabilities via effective remyelination and the prevention of subsequent neurodegeneration [3,4]. Among the cells that are transplanted, oligodendrocyte progenitor cells (OPCs) are particularly important because they can create new myelinating OLs, which help repair damaged myelin [5,6]. OPCs make up 5–8 percent of all the cells in an adult brain and can be found in every MS lesion, including the edges of chronic demyelinated plaques, but they remain quiescent and undifferentiated [7]. Following CNS damage, signaling from microglia and astrocytes activates OPCs from their quiescent state [8]. However, their numbers decrease with age and disease duration [9]. Also, the reduced capacity of OPCs, such as less proliferation and differentiation, and inadequate migration of OPCs into lesions, along with changes in extracellular matrix (ECM) components, caused failure in myelin regeneration [10,11]. Thus, boosting OPC populations may serve as an initial strategy to enhance remyelination in the CNS. Sun et al. demonstrated OPCs derived from mouse embryonic stem cells (mESCs) improved remyelination of spinal cord injury at the cervical zone and forelimb locomotor function [12]. In other research, Chen et al. indicated OPC transplantation formed a myelin sheath in periventricular leukomalacia brain injury [13]. However, the studies did not assess the impact of the prior microenvironment on isolated OPC or OPC effects in the newly transplanted microenvironment. Furthermore, the regulatory extracellular matrix component, as the cellular microenvironment, influences the signaling and cell interactions, differentiation, and migration of OPCs; its alterations are implicated in remyelination failure [14]. For instance, chondroitin sulfate proteoglycans (CSPGs), an important part of the ECM, are secreted by activated astrocytes following tissue injury [2]. CSPGs are a large family characterized by a protein core and repeating glycosaminoglycan sugar units, which exhibit structural variations due to sulfation at various sites [15]. CSPG upregulation in MS, such as versican, aggrecan, and neurocan, plays an inhibitory role for OPC migration, differentiation to myelinated OLs, and remyelination failure [16,17]. On the other hand, the cuprizone toxin model causes changes like astrogliosis and microgliosis, primary oligodendrogliopathy, atrophy in the corpus callosum, an intact blood-brain barrier (BBB), and a change of CSPG content, resulting in widespread loss of myelin in certain brain regions (pattern III MS lesions), which fits with a progressive type of MS [18,19]. Consequently, it serves as a simple, reproducible, and useful animal model for evaluating the remyelination process. Although it takes time to achieve chronic demyelination [20]. A notable aspect of the chronic cuprizone model after stopping toxin feeding is that, while remyelination occurs, its extent is significantly diminished [21].

The aim of this study is to investigate the effect of the previous microenvironment on the remyelinating capacity of OPCs isolated from two types of inflammatory and non-inflammatory microenvironments after transplantation into a new chronic non-inflammatory microenvironment of cuprizone.

## Materials & methods

### Study design

30 mature male C57BL/6 mice (approximately 20–25 g; 8–10 weeks) were purchased from the Pasteur Institute of Karaj (Iran). For adaptation, the animals were maintained in a regulated conditions with unlimited food and water and a standard 12 h light/dark cycle for one week. The clinical signs of the mice, including weight loss, dehydration, lethargy and decreased activity, and neurological impairments, were monitored daily. Body weight was recorded weekly during the experiment. All animals survived until the end of the experiment. After the designed time, the animals were anesthetized with ketamine/xylazine to reduce suffering and pain and then euthanized. All procedures were performed by trained professionals. The Tehran University of Medical Science Institutional Animal Care and Use Committee (IACUC) approved all animal-related procedures (ethics number: IR.TUMS.AEC.1402.120).

In the first step, 30 mice were randomly divided for two purposes:

1. 6 mice were used to isolate OPCs from the cuprizone (CPZ) model (C-OPCs) and lipopolysaccharide (LPS) model (L-OPCs) (n = 3 from each model).
2. 24 mice were classified into 3 groups (n = 8 per group): (1) CPZ group (the chronic cuprizone demyelination that was considered as the control group), (2) CPZ + C-OPCs group (the chronic cuprizone demyelination that received transplanted OPCs isolated from the CPZ model (C-OPCs)), and (3) CPZ + L-OPCs group (similar to the second group, but received OPCs isolated from the LPS model (L-OPCs)).

### Demyelination model

Part 1: The OPCs’ isolation was performed by activating them from a quiescent state. Consequently, two models were selected to be activated: 1. Cuprizone model-induced non-inflammatory demyelination 2. Lipopolysaccharide model-induced neuroinflammation demyelination [22]. A multiple sclerosis model was designed, following previous research:

1. Cuprizone model: Mice received a powdered diet chow mixed with 0.2% (W/W) cuprizone (Sigma-Aldrich, St. Louis, MO) for 12 weeks to achieve chronic demyelination [23].
2. Lipopolysaccharide model: Mice were injected with LPS (1 mg/kg body weight) intraperitoneally (Escherichia coli O55:B5, L2880 10 mg; Sigma-Aldrich, St. Louis, MO), a single time per day for five days to induce a model of chronic neuroinflammation [24].

Part 2: Finally, the chronic cuprizone model was utilized, as described in Part 1 above, for cell transplantation in the 13th week, and subsequently, the mice were fed a typical diet for 1 week. Then we examined the objectives of this study.

### Isolation, culture, and purification of OPCs

The procedure for acquiring OPCs has been explained and is based on previous research methods [25,26]. In summary, the brains of mice in the LPS and CPZ models were extracted after cervical dislocation and situated in a sterile petri dish with chilly HBSS. The brain’s meninges were eliminated, and the tissue was cut into 2 mm^3^ pieces. Digestion was facilitated by pipetting with trypsin and collagenase IV followed by an incubation at 37°C. The pellet was suspended in OPCs medium (high-glucose Dulbecco’s modified Eagle’s medium (DMEM; Gibco, USA) and 10% fetal bovine serum (Biochrom, Germany) and 1% penicillin/streptomycin (Sigma‐Aldrich)) and cultured in T25 cm^2^ poly-L-lysine-covered flasks with OPCs medium supplemented with PDGF AA (10 ng/ml; 100-13A, PeproTech) for 15 days. After an additional 10 days in the OPC medium without supplement, a mixed culture of OPCs, astrocytes, and microglia was achieved. To obtain pure OPCs, the flasks were shaken for 18–20 hours at 37°C and 250 rpm and then cultured on an uncoated flask for 45 minutes. This procedure allowed microglial adhesion but prevented OPCs from adhering. The collected OPCs were cultured in OPC medium for one week again, and finally, we used a high purity of OPCs for subsequent steps.

### Immunocytochemistry (ICC)

The identified OPCs were verified using the primary and secondary antibodies mentioned as follows: rabbit anti-PDGFRα (platelet-derived growth factor receptor α) (Abcam; ab203491), goat anti-rabbit FITC conjugated (Elabscience; E-AB-1014), mouse anti-Olig2 (oligodendrocyte transcription factor 2) (Santa Cruz; sc-518069), and goat anti-mouse CY3 conjugated (Elabscience; E-AB-1011). The cells were fixed with 10% formalin for 2 hours and washed twice and treated with Triton X-100 and BSA. Primary antibodies were incubated with the cells overnight, followed by secondary antibodies for 10 minutes. The nuclei were stained with DAPI. The cells were mounted and covered. Images of OPCs were captured by a fluorescence microscope (Olympus BX50, Japan) equipped with a camera (Olympus DP72, Japan) [27]. PDGFRα and Olig2 were quantified by analyzing the immunostained regions with ImageJ software. The results were exhibited as the percentage of positively staining regions.

### DiI labeling of OPCs, transplantation, homing assay

After counting the gathered OPCs, 1 _×_ 10^6^ cells were labeled with 2 μg DiI/mL according to the manufacturer’s instructions (Life Technologies, USA). Injection of 3 × 10^5^ OPCs in 100 µL PBS [28] was performed via the tail vein for the mice of the CPZ + C-OPCs and CPZ + L-OPCs groups. The homing of transplanted OPCs was evaluated in the corpus callosum and in visceral organs (lung, liver, spleen) after 24 h. For histological assessments, ketamine (Sigma, St. Louis, USA) and xylazine (Heidelberg, Germany) were injected intraperitoneally (IP) to completely anesthetize the mice. After perfusing the mice with a 0.9% NaCl solution and 4% paraformaldehyde (PFA, Merck, Germany), the tissues were extracted and placed in 10% formalin for 48 h. Sections of 5 μm thickness were obtained from the tissues. The nuclei were stained with DAPI. Images of sections were captured by a fluorescence microscope (Olympus BX51, Japan) equipped with an E.30 digital camera (Olympus, Japan) [29,30]. We quantified the percentage of arriving OPCs in the corpus callosum by using the ImageJ cell counter plugin to count DiI-labeled cells and divide them by the whole number of cells multiplied by 100. Additionally, we counted the arriving DiI-labeled OPCs to the lung, liver, and spleen per field by using the ImageJ cell counter plugin.

### Luxol fast blue (LFB) staining

The study used Luxol Fast Blue (LFB) staining to identify the demyelinating CPZ model and assess the remyelinated zone in the corpus callosum based on the method of previous research [31]. Randomly, 10 sections per mouse were captured by a light microscope (Olympus CX310, Japan) equipped with an E.30 digital camera (Olympus, Japan). The healthy mice’s brains were used to compare the corpus callosum and the CPZ model. The myelinated zone was quantified using ImageJ software, and the percentage was determined by dividing the LFB-dyed region by the total chosen region multiplied by 100.

### Immunofluorescence (IF) staining

After OPC transplantation, the CSPG4 in the microenvironment of the corpus callosum was verified using the primary and secondary antibodies mentioned as follows: mouse anti-CSPG4 (Santa Cruz; sc-166251) and goat Anti-Mouse (Abcam; ab6785). The sections were prepared and histologically examined, with antigen retrieval, heating, and incubation with 1% BSA in TBS for 2 hours. The sections were then incubated with primary antibodies overnight, followed by secondary antibodies for 10 minutes. The nuclei were stained with DAPI. The sections were mounted and covered. Images of the corpus callosum were captured by a fluorescence microscope (Olympus BX50, Japan) equipped with a camera (Olympus DP72, Japan) [32]. CSPG4 was quantified by analyzing the immunostained regions with ImageJ software. The results were exhibited as the percentage of positively staining regions.

### Quantitative real-time PCR

mRNA expression assessment was conducted on liquid nitrogen-frozen corpus callosum tissue using RT-qPCR for SRY-Box Transcription Factor 10 (SOX10), Myelin Basic Protein (MBP), Myelin Oligodendrocyte Glycoprotein (MOG), and Transforming Growth Factor β (TGFβ). All procedures followed the kit instructions. Extraction of total RNA was performed with SinaPure RNA (Sinaclon, Iran) and quantified with a NanoDrop 1000 (Thermo Fisher Scientific). Synthesis of cDNA was performed with the Easy cDNA Synthesis Kit (Parstous, Iran). VERNER SYBER Green Master Mix (Thermo Fisher Scientific) was combined with equal amounts of cDNA and designed primers in a StepOnePlus real-time PCR system (Applied Biosystems, USA). The sequences of the primer are as follows: SOX10, F:GACGZTGACAAGTTCCCCGT and R:TCCTCAATGAAGGGGCGCTT; MBP, F:GTCCCTGAGCAGATTTAGCTT and R:GAATCCCTTGTGAGCCGATT; MOG, F:CAGATGAAGGAGGCTACACC and R:GCACGAAGTTTTCCTCTCAGTC; TGFβ, F:GAACCAAGGAGACGGAATAC and R:AGTTGGTATCCAGGGCTCTC. The ΔCT method quantified gene expression relative to the housekeeping gene (β-Actin), and CPZ groups (as the control group) were employed for normalizing the data [32].

### Statistical analysis

The analysis of data was performed by GraphPad Prism Software (version 7, Boston, MA, USA) and displayed as mean ± SD. Shapiro-Wilk tests confirmed that the data distribution was normal. Unpaired T-tests and one-way ordinary ANOVAs followed by Tukey post hoc tests, in order, were utilized to compare two and three groups. The P-value of less than 0.05 was regarded as significant. * P < 0.05, ** P < 0.01, and *** P < 0.001 indicate significance.

## Results

### Characterization of OPCs

**Fig 1:**
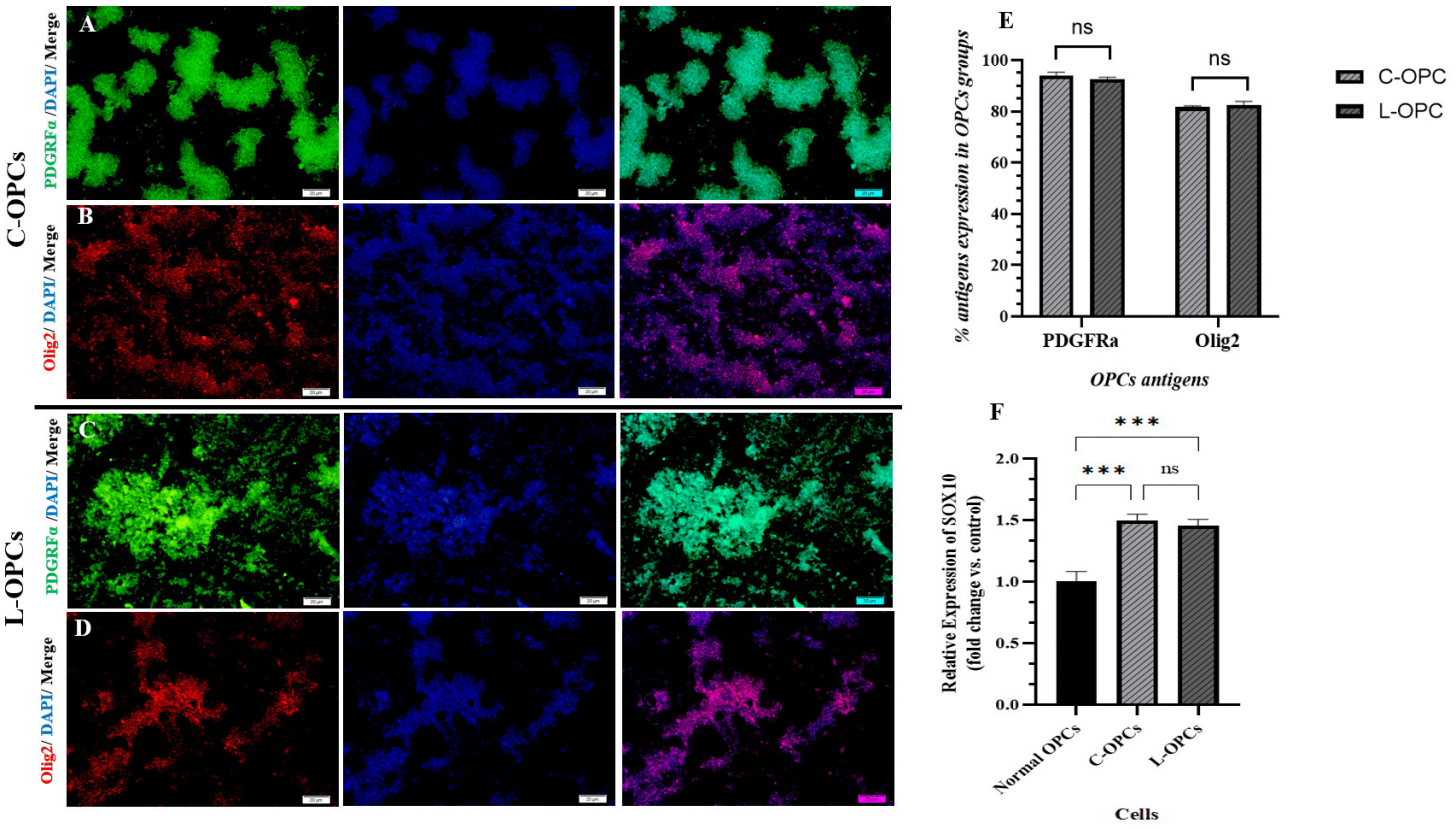
Characterization of OPCs by immunocytochemistry. **(A)** PDGRFα expression in C-OPCs, **(B)** Olig2 expression in C-OPCs, **(C)** PDGRFα expression in L-OPCs, **(D)** Olig2 expression in L-OPCs (scale bar = 20 µm). **(E)** Chart of quantitative analysis of PDGFRα and Olig2 markers in isolated OPCs from the CPZ and LPS model (n = 3), **(F)** Chart of quantitative analysis of SOX10 expression in the normal OPCs and isolated OPCs from the CPZ and LPS model (n = 3). Means ± SD are used to define all data. (ns: non-significant); *P < 0.05, **P < 0.01, and ***P < 0.001.

For purified isolated OPCs before transplantation, an immunocytochemistry was performed to measure the expression of two specific OPC markers, PDGFRα and Olig2 (Fig 1A-1D). The analysis results in the chart showed that the percentages of PDGFRα were (94.1 ± 1.16) and (92.6 ± 0.660) respectively, in OPCs isolated from CPZ models and LPS models. The analysis results in the chart showed that the percentages of Olig2 were (81.7 ± 0.533) and (82.6 ± 1.37), respectively, in OPCs isolated from CPZ models and LPS models (Fig 1E). To further characterize the isolated OPCs, the expression of the transcription factor SOX10 by RT-qPCR was significantly increased in both C-OPCs (1.50 ± 0.04) and L-OPCs (1.46 ± 0.05) compared to the normal OPCs (1.00 ± 0.08) (p < 0.001 for both comparisons) (Fig 1F). However, the difference in PDGFRα, Olig2, and SOX10 expression between the C-OPCs and L-OPCs was not statistically significant (p > 0.05). The strong expression of specific markers of OPCs approved the identity of the isolated cells as OPCs.

### Homing assay

**Fig 2:**
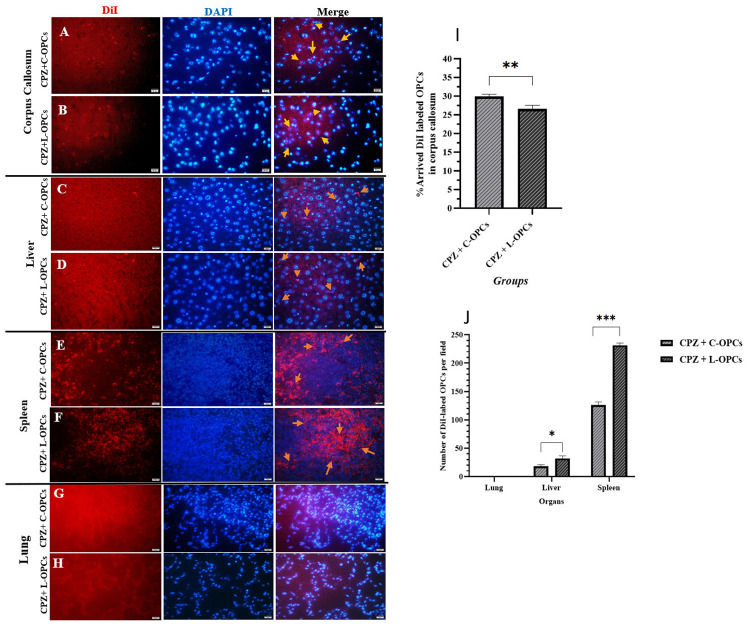
DiI-labeled OPCs in the corpus callosum and visceral organs (lung, liver, and spleen) 24 h after transplantation. Results of the CC showed C-OPC homing was higher than L-OPCs: **(A)** CC of the CPZ + C-OPCs group, **(B)** CC of the CPZ + L-OPCs group (scale bar = 50 µm). Also, results of the liver and spleen showed trapped C-OPCs were less than L-OPCs: (C) liver of the CPZ + C-OPCs group, (D) liver of the CPZ + L-OPCs group, (E) spleen of the CPZ + C-OPCs group, and (F) spleen of the CPZ + L-OPCs group (scale bar = 50 µm). No OPCs showed in the lung: (G) lung of the CPZ + C-OPCs group, (H) lung of the CPZ + L-OPCs group (scale bar = 50 µm). **(I)** Chart of quantitative analysis of homing OPCs in the CC (n = 4). **(J)** Chart of quantitative analysis of trapped OPCs in the lung, liver, and spleen (n = 3). Means ± SD are used to define all data. *P < 0.05, **P < 0.01, and ***P < 0.001.

After transplantation, DiI-labeled OPCs were evaluated in mouse models. The results revealed that the corpus callosum contained two types of isolated OPCs 24 h after transplantation. In particular, the percentage of C-OPCs in the CPZ + C-OPCs group (29.9 ± 0.634) was significantly greater than the percentage of L-OPCs in the CPZ + L-OPCs group (26.6 ± 0.986; p < 0.01). This result indicates that C-OPCs had a greater capacity for migration or survival rate within the corpus callosum than L-OPCs (Fig 2A-2B). The chart (Fig 2I) also represents these results. Further analysis of the visceral organs revealed the presence of two types of transplanted OPCs in the liver and spleen 24 h after transplantation. However, the number of C-OPCs per field in the liver and spleen of the CPZ + C-OPCs group (18.0 ± 3.00 and 126 ± 5.57, respectively) was significantly less than the number of L-OPCs in the CPZ + L-OPCs group (32.0 ± 4.58 and 231 ± 4.04, respectively) (p < 0.05 and p < 0.001, respectively). These results indicated that C-OPCs were less likely to be trapped in the liver and spleen, which may explain their greater presence in the corpus callosum. No isolated OPCs were observed in the lung for either group (Fig 2C-2H). The chart (Fig 2J) also represents these results.

### LFB staining for remyelination

**Fig 3:**
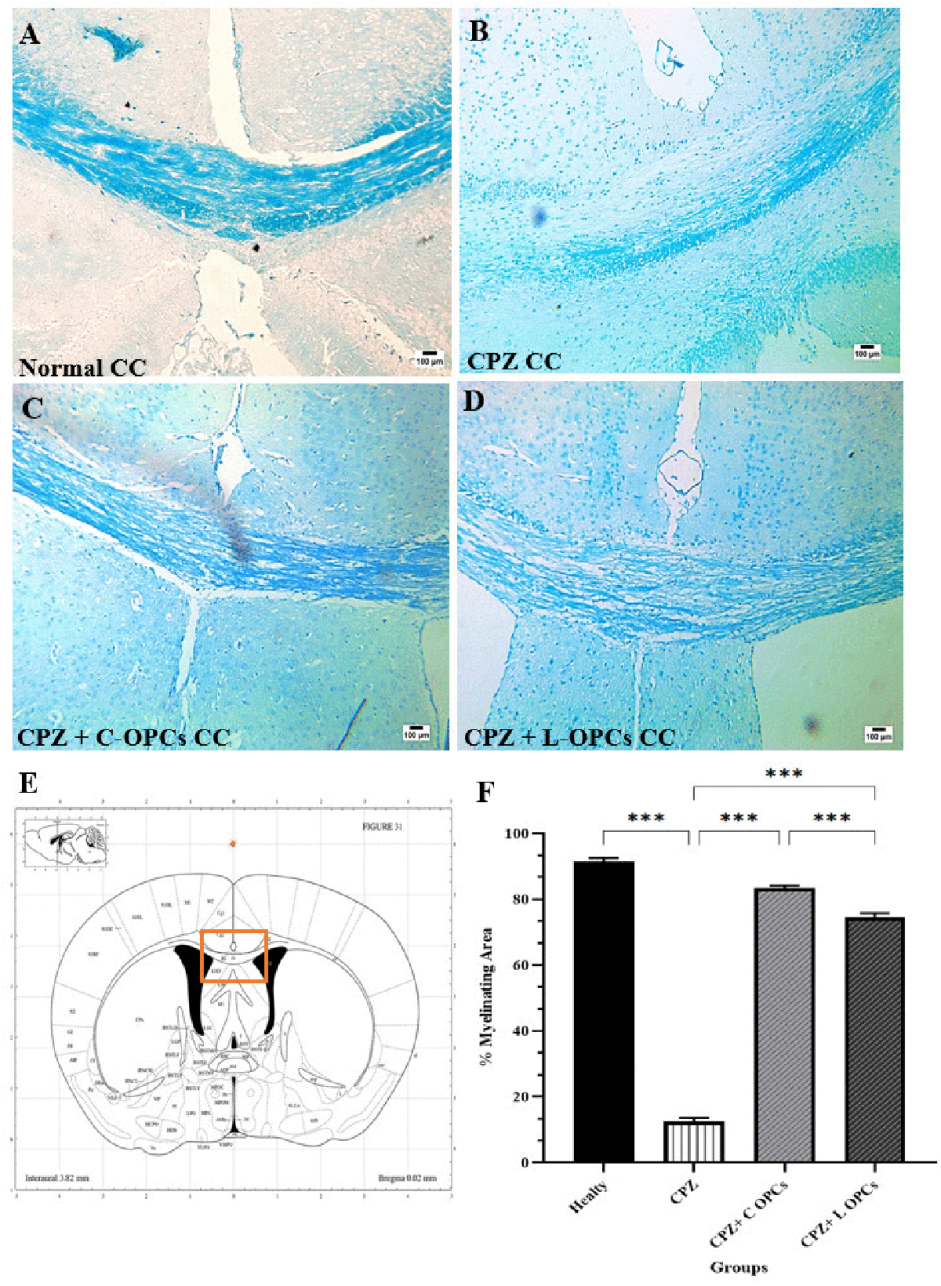
Evaluation of myelinating area in the corpus callosum by LFB staining. Results showed complete myelin content (blue color) in healthy and decreased myelin content in the chronic CPZ group (white color); after OPC transplantation, the myelin content increased in CPZ + C-OPCs & CPZ + L-OPCs; also, the increase was higher in CPZ + C-OPCs; **(A)** Healthy CC, **(B)** CC of Chronic CPZ group, **(C)** CC of CPZ + C-OPCs group, **(D)** CC of CPZ + L-OPC group (scale bar = 100 µm), **(E)** Mouse coronal brain atlas of Paxino. **(F)** Chart of quantitative analysis of the remyelination area in the corpus callosum for all groups (n = 3). Means ± SD are used to define all data. *P < 0.05, **P < 0.01, and ***P < 0.001.

According to the LFB staining assessment, the corpus callosum of the healthy group exhibited normal myelin content (blue) (91.5 ± 1.06). On the other hand, the corpus callosum of the CPZ group showed considerable demyelination (white) (12.4 ± 1.22) or a significant reduction in the myelinated area compared to the healthy group (p < 0.001). The corpus callosum in the CPZ + C-OPCs (83.4 ± 0.849) and CPZ + L-OPCs (74.5 ± 1.29) groups displayed a considerably higher myelin content (p < 0.001) as compared to the CPZ group. This indicates that the remyelination after OPC transplants was significantly improved. The CPZ + C-OPCs group showed better myelin content than the CPZ + L-OPCs group, which indicates C-OPCs had a more significant remyelination improvement (p < 0.001) (Fig 3A-3E). The chart (Fig 3F) also represents these results.

### IF staining for CSPGs

**Fig 4:**
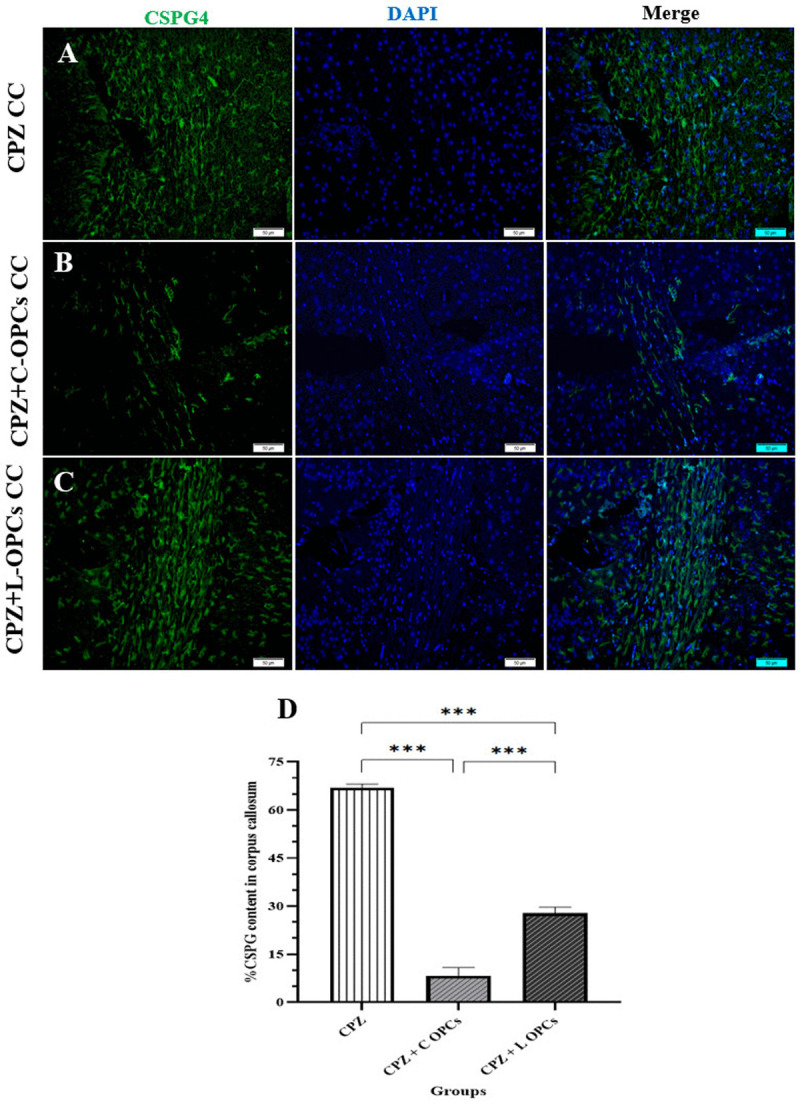
Evaluation of CSPG4 level in the corpus callosum by IF. Results showed an increase in the CPZ group; also, after transplantation, CSPG4 decreased in both the CPZ + C-OPCs group and the CPZ + L-OPCs group but significantly more in the CPZ + C-OPCs group compared with others: (A) chronic CPZ group, **(B)** CPZ + C-OPCs group, **(C)** CPZ + L-OPCs group (scale bar = 50 µm). **(D)** Chart of quantitative analysis of CSPG level in the corpus callosum for all groups (n = 3). Means ± SD are used to define all data. *P < 0.05, **P < 0.01, and ***P < 0.001.

Immunofluorescence staining for CSPG4 showed significant changes in its levels within the corpus callosum. CSPG4 levels were high in the demyelinated CPZ group (67.0 ± 1.14), reflecting the inhibitory microenvironment that typically forms after injury in MS. In contrast, the CSPG4 levels for the CPZ + C-OPCs group and the CPZ + L-OPCs group were (8.20 ± 2.65 and 27.8 ± 1.88, respectively, which indicates both the CPZ + C-OPCs and CPZ + L-OPCs groups showed a significant decrease in CSPG4 levels compared to the CPZ group (p < 0.001). This reduction suggests that the transplanted OPCs were enabled to modulate the inhibitory environment. Furthermore, a direct comparison between the two treatment groups showed that the decrease in CSPG4 levels was significantly greater in the CPZ + C-OPCs group than in the CPZ + L-OPCs group (p < 0.001). This evidence indicates that C-OPCs were more effective at reducing CSPG4 levels and, by extension, had a greater positive impact on the microenvironment, which aligns with the above remyelination results observed with C-OPC transplantation (Fig 4A-4C). The chart (Fig 4D) also represents these results.

### Expression of MOG, MBP, and TGFβ

**Fig 5:**
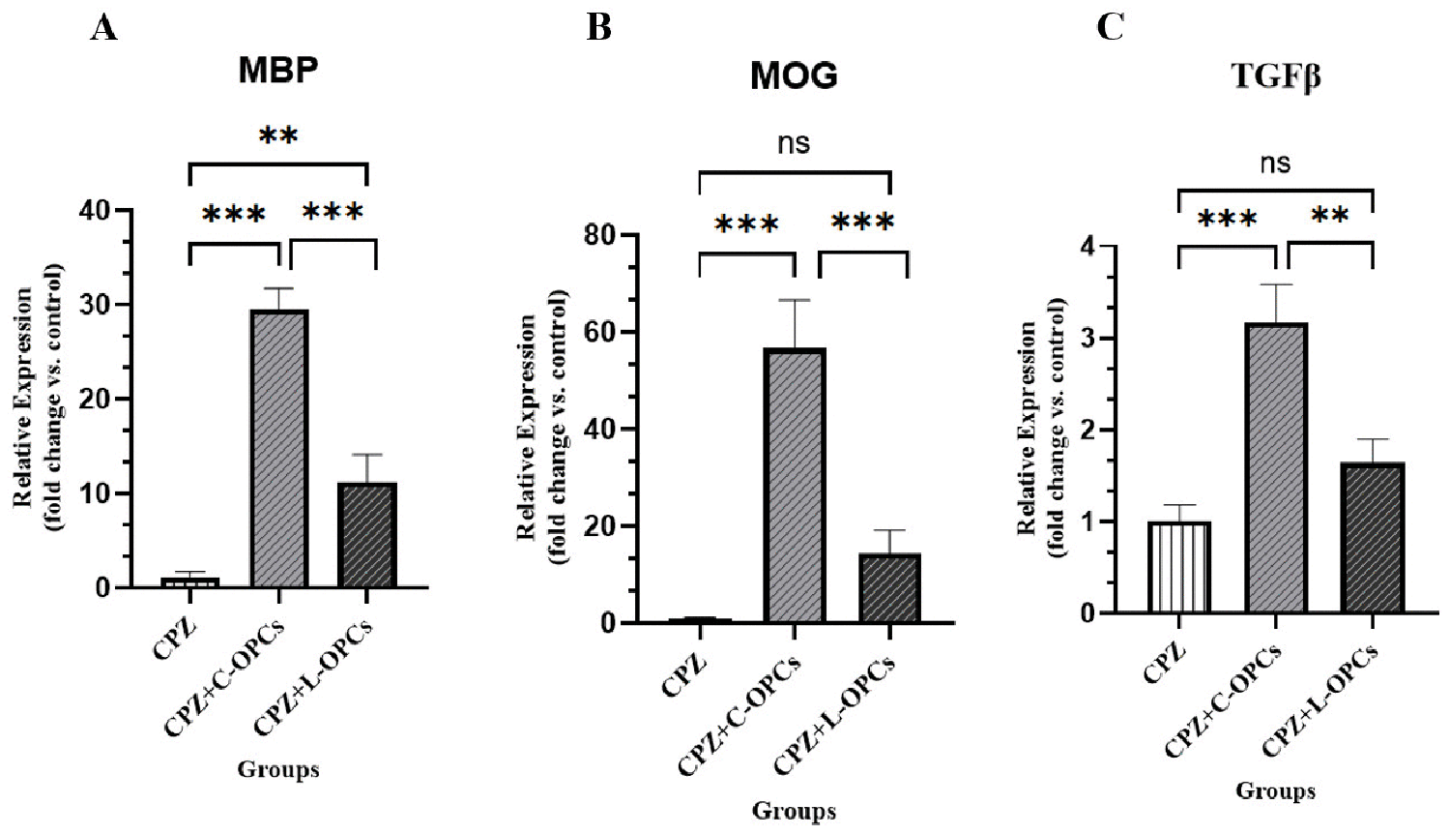
Evaluation of (A) MBP, (B) MOG, and (C) TGFβ mRNA expression by RT-qPCR and quantitative analysis. Results showed significantly higher levels in the CPZ + C-OPCs group compared with the CPZ & CPZ + L-OPCs groups (n = 3). Means ± SD are used to define all data. (ns: non-significant); *P < 0.05, **P < 0.01, and ***P < 0.001.

RT-qPCR analysis of MBP expression, as a crucial component of the myelin sheath, indicated considerable enhancements in remyelination. The CPZ group exhibited a significantly reduced MBP expression (1.10 ± 0.566), demonstrating severe demyelination. Conversely, the CPZ + C-OPCs group (29.6 ± 2.15) and the CPZ + L-OPCs group (11.2 ± 2.92) exhibited a significant elevation in MBP expression compared to the CPZ group (p < 0.001 and p < 0.01, respectively). Therefore, both types of transplanted OPCs enabled new remyelination. The increase of MBP expression was significantly higher in the CPZ + C-OPCs group than in the CPZ + L-OPCs group (p < 0.001), which means that C-OPCs were more effective at improving remyelination (Fig 5A). We also evaluated the expression of MOG, which is another important myelin protein. The MOG expression was reduced in the CPZ group (1.01 ± 0.178). A significant increase of MOG expression was detected in the CPZ + C-OPCs group (56.8 ± 9.81) compared to the CPZ group and the CPZ + L-OPCs group (14.3 ± 4.85) (p < 0.001 for both comparisons). Notably, the increase of MOG expression in the CPZ + L-OPCs group was not significant compared to the CPZ group (p > 0.05). This finding supports the enhanced remyelinating capacity of the C-OPCs (Fig 5B). Finally, RT-qPCR was used to evaluate the expression of an important cytokine, TGFβ. The CPZ + C-OPCs group showed a significant increase in TGFβ expression (3.17 ± 0.411) compared to both the CPZ group (1.01 ± 0.173) and the CPZ + L-OPCs group (1.64 ± 0.259) (p < 0.001, p < 0.01), respectively. The increase in TGFβ expression in the CPZ + L-OPCs group was not significant when compared to the CPZ group (p > 0.05) (Fig 5C), which has been discussed in detail.

## Discussion

Our study showed that transplantation of OPCs isolated from both inflammatory and non-inflammatory microenvironments simultaneously into the chronic cuprizone demyelination model facilitated remyelination; however, myelination was significantly greater in the CPZ + C-OPCs group than in the CPZ + L-OPCs group. Increased expression of MBP and MOG genes further confirmed these results. Also, transplantation of OPCs isolated from both inflammatory and non-inflammatory microenvironments resulted in a decrease in CSPG, which was more significant in the CPZ + C-OPCs group compared to the CPZ + L-OPCs group.

Consistent with prior studies, CPZ serves as a model in animal research to examine demyelination and remyelination processes, as well as therapeutic strategies for MS, without damaging the BBB or causing peripheral immune system stimulation or adaptive autoimmune responses, and it is regarded as a simple, reproducible model [20,33,34]. Feeding cuprizone for 12 weeks, as a model of chronic demyelination, results in the destruction of mature OLs due to copper deficiency in complex IV of the respiratory chain and mitochondrial dysfunction [35]; it diminishes their ability to preserve existing myelin and ultimately leads to significant demyelination of the corpus callosum [36]. Conversely, following the termination of cuprizone treatment, despite a decrease in the reserve of OPCs, spontaneous remyelination occurs at a rate of under 30% but requires at least 9 weeks [37,38]. Thus, the chronic cuprizone model can be appropriate and cost-effective for examining the impact of cell transplantation isolated from various isolation microenvironments.

Recent studies have shown that transplantation of various cells (including embryonic stem cells (ESCs), induced pluripotent stem cells (iPSCs), mesenchymal stem cells (MSCs), neural progenitor cells (NPCs), and oligodendrocyte progenitor cells (OPCs)) in animal and pathological demyelination models has improved remyelination by replacing lost cells and modulating the immune system [39,40]. Among them, OPCs have received more attention for their differentiation into myelinating oligodendrocytes, thereby reducing OL and neuronal death, neuroprotection, and ultimately improving remyelination [13,41]. In a study, Chen et al. indicated transplanted OPCs in periventricular leukomalacia (PVL), a prevalent ischemic brain injury, persevered and generated a myelin sheath and stimulated neural stem cell (NSC) proliferation [13]. Bojnordi et al. demonstrated that bone marrow stromal-derived OPC transplantation into the rat corpus callosum improved remyelination in a lysophosphatidylcholine-induced (LPC) demyelination model in contrast with the control group [42]. Fan et al. revealed that transplantation of OPCs derived from miR219-mESCs (miR219-OPCs) promoted remyelination in a chronic cuprizone model in C57BL/6 male mice, improved cognitive capacity, and enhanced the endogenous NPC proliferation [43]. Therefore, in this study, OPCs, which are the progenitor cells of myelinating OLs, were used. However, prior studies have not reported on the role of the microenvironment of OPC isolation and its effect on their differentiation and myelination capacity post-transplantation.

Cells at each stage of oligodendroglia lineage maturation and differentiation, from OPCs to myelinating OLs, express specific and distinct morphology and markers. OPCs are identified by markers, including specific mitogenic receptors as platelet-derived growth factor receptor (PDGFRα), proteoglycan NG2, ganglioside A2B5, or transcription factors SOX10 and OLIG2 [44,45]. It has been observed that signaling through the binding of platelet-derived growth factor α (PDGFα) and the PDGFRα ensures the proliferation and survival of OPCs [46,47]. In other hand, OPCs express SOX10 [48], which is crucial for activating and differentiating OPCs into myelinated oligodendrocytes and directly activating myelin genes [49]. So, increased SOX10 plays a critical role in determining oligodendroglia fate [50]. Also, increased SOX10 significantly increases the expression of Olig2 in OPCs [51]. Increased OLIG2, in turn, acts to increase OPC migration and increase remyelination [52]. Notably, The expression of Olig2 and Sox10 indicates the pre-OPC stage, while the presence of PDGFRα indicates progression to the OPC stage [53]. Therefore, the factors PDGFRα, Olig2, and SOX10 were considered as specific markers characterizing OPC before transplanting, and confirmed by both C-OPC and L-OPC groups, without significant differences.

In our study, it was observed that after intravenous injection, under similar transplantation conditions, both non-inflammatory (C-OPC) and inflammatory (L-OPC) OPCs were engrafted and established in the corpus callosum. This is because OPC migration is modulated by different molecules, both during the development of the central nervous system and after demyelination in the adult central nervous system [54]. For example, CCl2 and IL1 are chemoattractant factors that are secreted by inflammatory cells, microglia, and astrocytes in neurological diseases such as Alzheimer’s and MS (both in acute and chronic lesions). These factors increase their motility and migration by affecting their receptors (CCR2 and ILR1) in activated OPCs [55]. On the other hand, increased expression of CCL2 has been observed in the cuprizone model [56] (the host tissue of the study), which itself causes more C-OPCs to be attracted to the demyelinated lesions of the corpus callosum of the brain. However, it has been observed that in inflammatory models of MS, attractant chemokines such as CCL2 and CCL5 undergo structural and mitogenic changes [57], and perhaps this could explain the role of less L-OPCs recruitment to the corpus callosum by negatively affecting cellular receptors. However, our data do not allow further explanation in this regard.

Neuroinflammation induced by LPS can damage the BBB, causing LPS, together with inflammatory cells and their components, to reach the brain [58]. LPS also promotes the production of inflammatory cytokines, IL-1β, IL-6, and TNF-α, from immune cells [59]. Also, in the cuprizone model, microglia accumulation leads to an increase in pro-inflammatory factors IL-1α and IFN-γ, but after cuprizone cessation, these factors decline, followed by an increase in the anti-inflammatory cytokine IL-10 [60]. In a study by Omri Zveik et al., after co-culturing OPC in vitro with anti-inflammatory factors interleukin IL-4 and IL-10, OPC differentiation was reduced, and with pro-inflammatory factors interferon (IFN)-γ and tumor necrosis factor (TNF)-α, the harmful effect of interferon was eliminated and differentiation was increased [61]. Another investigation showed increased differentiation of OLs in an environment with microglia treated with anti-inflammatory cytokines (IL10 and IL13) [62]. So, according to our DiI and LFB observations, for the first time after transplanting OPCs isolated from two types of microenvironments in vivo, L-OPCs had a lower rate of homing and remyelination in the corpus callosum compared to the C-OPC group. They were also more likely to be trapped in lymphoid organs such as the spleen and liver.

TGFβ is a multifunctional cytokine that has diverse functions, including cell differentiation and migration, growth promotion and inhibition, and immune system modulatory effects [63]. According to prior research, OPCs secrete transforming growth factor β (TGFβ), which affects the cerebral endothelium via activating the MEK/ERK pathway and then improves the blood-brain barrier (BBB) integrity, which is a factor in healing [64]. Also, OPCs suppress microglia activation and provide immune homeostasis in the brain for myelin regeneration through the TGFβ2- TGFβ type II receptor (TGFβR2)-CX3C chemokine receptor 1 (CX3CR1) signaling chain [65,66]. Additionally, the TGFβ signaling pathway suppresses inflammation via influences on Treg cell activity and influences remyelination [67]. The TGFβ-TGFβR signaling mechanism in OPC cells modulates and activates the Smad2/3/4-FoxO1-Sp1cascade complex. This signaling cascade in turn inhibits and activates c-myc and p21 transcription, respectively, which ultimately causes OPCs to exit the cell cycle and progress toward myelinating oligodendrocyte differentiation at the appropriate time and remyelination [68]. Other observations showed that TGFβ1 administration increased remyelination in an animal model of MS, and its reduction prevented myelin regeneration in demyelinated spinal cord [69]. However, another study stated that increased TGFβ levels due to aging and microglia dysfunction caused a decrease in OPC differentiation [70]. However, it is noteworthy that our results also showed that due to the increased expression of TGFβ in the CPZ + C-OPC group compared to the other groups, myelination was also increased in this group.

In the CNS, myelin basic protein (MBP) is generated by myelinating OLs, and it is crucial for the myelin sheath’s compactness and rigidity [71]. Myelin oligodendrocyte glycoprotein (MOG), a minor component, is essential to maintain the integrity of the myelin sheath and act as an important communication part in the myelin sheath [72]. Research shows that MBP and MOG reduction is significant after 1 week of CPZ feeding [34]. In our chronic cuprizone model, a severe decrease in these proteins was observed after 12 weeks; however, following transplantation, there was a significant increase in these proteins in the CPZ + C-OPCs group compared to the CPZ + L-OPCs and CPZ groups, confirming greater improvement in myelination.

Prior studies showed that high expression of CSPG by reactive astrocytes causes increased stiffness of ECM in the chronic cuprizone model and inhibition of remyelination [73]. Besides, mechanical sensitivity of OPCs to the microenvironment prevents cell migration and differentiation, reducing the remyelination process [74,75]. Research methods indicated that reducing CSPG may enhance remyelination. As an example, Nori et al. showed that simultaneous NPC transplantation and chondroitinase ABC in a rat spinal cord injury model improved NPC survival, differentiation into OLs, remyelination, and nerve function recovery [76]. Luo et al. demonstrated that in an inflammatory EAE model of MS, inhibiting the binding of CSPG to its receptor, protein tyrosine phosphatase σ (PTP-σ), using the intracellular sigma peptide increased cell migration, differentiation, remyelination, and animal function [77]. On the other hand, studies have shown that increased TGFβ increases the expression of three types of CSPGs, neurocan, brevican, and aggrecan, through the activation of non-Smad signaling pathways and the PI3K–Akt–Mtor molecular cascade [78,79]. However, no study has yet reported the role of transplantation of cells isolated from different microenvironments on the CSPGs. In current research, we demonstrated that transplantation of OPCs causes a reduction of CSPG that previously, by the chronic cuprizone model, increased in the microenvironment of the mouse brain. The important point is that the reduction of CSPG was significantly greater in the CPZ + C-OPCs group, which finally resulted in more remyelination. This result was despite the fact that we observed an increase in TGFβ in the same group, and our results were contrary to previous studies in this field, and in our study, these two factors worked in concert.

The findings of this study can be attributed to the high compatibility of OPCs isolated from the cuprizone model with the host environment because the host environment matches the source of cell isolation. Future research will provide additional data. It is suggested that future studies: 1. Examine other CNS regions to assess progress in myelination. 2. Monitor animals for long periods of time and assess motor function. 3. Assess environmental inflammatory and anti-inflammatory factors, cell-secreted factors, and additional ECM components. 4. Compare results using alternative models. 5. Evaluate the optimal environment for remyelination after cell transplantation so that cells can act as both immunomodulators and pro-myelinators.

## Conclusion

The failure of myelin regeneration frequently results from the inability of OPCs to differentiate into myelinating OLs within the pathogenic environment of MS. Our study showed remyelination and a reduction of CSPG4 following the transplantation of OPCs, activated under both inflammatory and non-inflammatory conditions, into the corpus callosum of a chronic cuprizone model. The most significant modifications were linked to the cuprizone-dissected cells, which closely matched the host tissue. Therefore, the increased effectiveness of transplanting for enhancing remyelination demands greater attention to the cellular background and features of the host environment.

## Supporting information

S1 File Raw data-image analysis. (XLSX)

S2 File Raw data-PCR. (XLSX)
